# Pulmonary Sequelae of COVID-19: Focus on Interstitial Lung Disease

**DOI:** 10.3390/cells12182238

**Published:** 2023-09-08

**Authors:** Janet Johnston, Delia Dorrian, Dermot Linden, Stefan Cristian Stanel, Pilar Rivera-Ortega, Nazia Chaudhuri

**Affiliations:** 1 Interstitial Lung Diseases Unit, North West Lung Centre, Wythenshawe Hospital, Manchester University NHS Foundation Trust, Manchester M23 9LT, UKpilar.rivera-ortega@mft.nhs.uk (P.R.-O.); 2 Wellcome-Wolfson Institute for Experimental Medicine, School of Medicine, Dentistry and Biomedical Sciences, Queen’s University Belfast, Belfast BT9 7BL, UK; 3 Mater Hospital, Belfast Health and Social Care Trust, Belfast BT14 6AB, UK; 4 Faculty of Biology, Medicine and Health, University of Manchester, Manchester M13 9PL, UK; 5 School of Medicine, Magee Campus, University of Ulster, Northlands Road, Londonderry BT48 7JL, UK; nazia.chaudhuri@nhs.net

**Keywords:** post-COVID ILD, COVID-19, interstitial lung disease, residual lung abnormalities, pulmonary fibrosis

## Abstract

As the world transitions from the acute phase of the COVID-19 pandemic, a novel concern has arisen—interstitial lung disease (ILD) as a consequence of SARS-CoV-2 infection. This review discusses what we have learned about its epidemiology, radiological, and pulmonary function findings, risk factors, and possible management strategies. Notably, the prevailing radiological pattern observed is organising pneumonia, with ground-glass opacities and reticulation frequently reported. Longitudinal studies reveal a complex trajectory, with some demonstrating improvement in lung function and radiographic abnormalities over time, whereas others show more static fibrotic changes. Age, disease severity, and male sex are emerging as risk factors for residual lung abnormalities. The intricate relationship between post-COVID ILD and idiopathic pulmonary fibrosis (IPF) genetics underscores the need for further research and elucidation of shared pathways. As this new disease entity unfolds, continued research is vital to guide clinical decision making and improve outcomes for patients with post-COVID ILD.

## 1. Introduction

On 31 December 2019, the World Health Organization (WHO) recognized cases of ‘viral pneumonia’ in Wuhan, China, which was later identified as SARS-CoV-2 [1]. By 11 March 2020, the WHO had declared COVID-19 a global pandemic. As of 30 May 2020, over 5.5 million cases had been registered with over 350,000 deaths [2]. To date, there have been over 760 million cases of SARS-CoV-2 confirmed globally and more than 6.9 million COVID-19-associated deaths recorded [3]. Acute COVID-19 can range from asymptomatic infection to acute respiratory distress syndrome (ARDS) [4]. The acute life-threatening sequelae of SARS-CoV-2 have been substantially diminished through unprecedented platform trials and successful vaccine rollout [5,6,7]. However, post-acute sequelae of COVID-19 (PASC) are frequently reported, and dyspnea consistently ranks highest amongst them [8]. PASC frequently persists beyond 12 months and is associated with a lower quality of life, reduced working capacity, and increased all-cause mortality [9]. There is mounting concern about the potential to develop interstitial lung disease (ILD) due to evidence of fibrotic changes observed as early as three weeks after infection [10]. The ongoing impact of post-COVID ILD (PCILD) and the need for new targeted therapies presents a significant challenge, especially when considering the substantial indirect economic impacts of the pandemic.

### What Are Interstitial Lung Diseases?

Interstitial lung disease (ILD) encompasses a group of conditions characterised by inflammation or fibrosis of the lung parenchyma [11]. ILDs with a precipitant cause include connective tissue disease-related ILD (CTD-ILD), drug-induced ILD, and chronic hypersensitivity pneumonitis (HP). Those without a known cause are referred to as idiopathic interstitial pneumonias (IIPs), the most common of which is idiopathic pulmonary fibrosis (IPF) [11]. The multi-disciplinary team (MDT) meeting is the current gold standard for the diagnosis and management of ILDs, increasing diagnostic accuracy and confidence [12]. IPF typically affects older adults and is characterised by progressive lung fibrosis leading to symptoms of shortness of breath and chronic cough. The current standard of care treatment includes antifibrotic medication to slow down progression medication (Pirfenidone and Nintedanib) [13,14], symptom management, and lung transplant in selected cases. Interestingly, demographic characteristics that have been identified as risk factors for severe cases of COVID-19, including male sex, older age, and hypertension, are also known risk factors for IPF [15].

## 2. Why Are We Concerned about Post-COVID ILD?

Data from the early stages of the pandemic in Wuhan identified ground glass opacification (GGO), consolidation, and septal thickening as the predominant radiological findings during acute COVID-19 illness [16,17]. These findings are in keeping with an organising pneumonia pattern, which has been found to be the most common radiological pattern [18,19].

Longitudinal studies of patients who survived previous outbreaks of coronaviruses, such as severe acute respiratory syndrome (SARS) and Middle East respiratory syndrome (MERS), found that a significant proportion had persistent chest radiographic abnormalities at 3 months and beyond, including reticulation [20,21]. Studies looking at longer-term effects of SARS on computerised tomography (CT) imaging found persistent interstitial lung abnormalities up to 7 and even 15 years post infection [22,23]. Based on this data, it is postulated that a sizable proportion of COVID-19 survivors will be left with persistent lung abnormalities.

In a study conducted during the early stages of the pandemic in Wuhan, fibrotic CT abnormalities were found as early as 56 days from symptom onset [24]. Interestingly, these fibrotic changes had a peripheral distribution, consistent with the predominant distribution of GGO and consolidation observed during the acute illness. Older age and acute illness severity were identified as risk factors for these fibrotic abnormalities [24].

Post-mortem studies of COVID-19 patients have shown that diffuse alveolar damage (DAD) is the predominant pattern of injury in the lungs [25,26]. DAD is characterised by damage to the alveolar-capillary barrier resulting in the accumulation of fluid and cells within the alveoli [27]. DAD has been shown in post-mortem studies of SARS and MERS patients, and it is also a characteristic histological feature of ARDS [28,29,30]. ARDS is defined by acute onset of hypoxemia and bilateral pulmonary opacities not fully explained by cardiac failure, with a PaO_2_/FiO_2_ (arterial oxygen pressure/fraction of inspired oxygen) ratio of less than 300 mmHg [31]. A retrospective study of 201 COVID-19 patients in Wuhan found that 41.8% developed ARDS [32], while a subsequent meta-analysis estimated the incidence at 14.8% [33]. DAD is not the sole pathological pattern observed in ARDS; however, it is associated with more severe cases and worse patient outcomes [28].

Approximately 25% of survivors of ARDS demonstrate a restrictive pattern of lung function at 180 days, and these patients also demonstrated fibrotic changes (reticular infiltrates) on CT imaging [34,35]. As post-ARDS pulmonary fibrosis is a well-established complication, its potential development as a long-term outcome of COVID-19 is a major concern [34,35]. The mechanisms responsible for the fibroproliferative process seen in ARDS are not fully understood, but current research suggests that it occurs due to the disruption of the alveolar-capillary barrier [35], which leads to the accumulation of fluid in the alveoli (alveolar oedema). This, in turn, triggers an acute inflammatory response characterised by the activation of pro-inflammatory cytokines and chemokines, which can cause further cellular damage [35]. If the damage is not repaired promptly, it can lead to a pathological fibroproliferative response [35]. It is thought that mechanical ventilation exacerbates the initial inflammatory injury seen in ARDS through the application of shear forces [35]. As a result, protective ventilation strategies, including the adoption of lower tidal volumes [36], have been extensively employed. However, the efficacy of these measures in mitigating the onset of lung fibrosis remains uncertain. Unlike IPF or other progressive ILDs, fibrosis resulting from ARDS tends to be more stable [34,35,37]. Whether PCILD will mirror the steady disease pattern observed in post-ARDS fibrosis or present a more alarming tendency toward progression is unclear.

## 3. Pathophysiology and Proposed Mechanisms of Post-COVID ILD

PCILD currently lacks a consensus definition, but several mechanistic similarities have been drawn between IPF and pulmonary fibrosis after severe COVID-19 (i.e., ARDS) [37]. Both induce apoptosis of alveolar epithelial type I + II cells (AEC), SARS-CoV-2 can directly infect AEC type II, leading to macrophage activation and a subsequent cytokine storm. This proinflammatory response causes AEC and endothelial cell damage, resulting in fibroblast activation and incorporation of a collagen-rich extracellular matrix in the interstitial space. In genetically susceptible individuals, the induction of profibrotic pathways has the potential to lead to pulmonary fibrosis [38].

### 3.1. Genome-Wide Association Studies (GWAS)

The heterogeneity in clinical responses to COVID-19 suggests that genetics may contribute to disease severity. COVID-19 and IPF share some similarities, such as a higher incidence in older males [15]. Genome-wide association studies (GWAS) have shed new light on the potential genetic aetiology of IPF identifying 20 genome-wide significant associations [39]. Aberrant mucin 5B, oligomeric mucus/gel-forming (MUC5B) expression has previously been strongly linked to IPF [40] and, more recently, Allen et al. reported that a common MUC5B promoter region variant, rs35705950, was associated with a five-fold increase in disease risk [41]. Interestingly, this same variant has been associated with a protective effect against COVID-19-related hospital admissions in older patients [42]. It remains unclear whether this apparently protective effect was partially explained by more stringent self-isolation in those at-risk. Conversely, further genes linked to IPF such as dipeptidyl peptidase 9 (DPP9) [43] have been independently reported to be associated with severe COVID-19 [44]. More recently the largest GWAS study comparing IPF and COVID-19 to date pooled data from five separate IPF studies demonstrating a significant but weak positive correlation between IPF and all COVID-19 severity phenotypes (*p* = 0.0045) [45]. This study reaffirmed previous findings that MUC5B, DPP9, and ATPase phospholipid transporting 11A (ATP11A) all represent shared genetic associations for both IPF risk and severity of COVID-19; however, crucially the analysis shows these findings are unified by a single causal variant [45]. In their conclusion, Allen et al. reported that the rs2897075_T allele at 7q22.1 signal co-localised with reduced levels of tripartite motif-containing protein 4 (TRIM4) and zinc finger with KRAB and SCAN domains 1 (ZKSCAN1) in the blood potentially paving the way for further mechanistic and translational studies targeting virus-induced interferon signalling pathways [45].

### 3.2. Profibrotic Macrophages in COVID-19: Implications for Lung Injury

Profibrotic CD163+ macrophages appear to be inextricably linked to the fibroproliferative response in the setting of SARS-CoV-2 [46]. Early studies by Liao et al. used single-cell RNA-sequencing demonstrated abundant proinflammatory monocyte-derived macrophages in bronchoalveolar lavage fluid (BALf) [47], while Szabo et al. reported an association between myeloid cells and mortality in COVID-19 [48]. Notably, the advent of spatial transcriptomics has enabled detailed evaluation of gene expression in situ following SARS-CoV-2 infection [49]. Margaroli et al. conducted a study comparing autopsy-derived lung tissue from patients with acute respiratory distress syndrome (ARDS) caused by Influenza A virus subtype H1N1 and SARS-CoV-2 infections [49]. The study found that SARS-CoV-2 infection was associated with a distinct transcriptional profile characterised by increased expression of genes related to epithelial-to-mesenchymal transition (EMT), coagulation, and extracellular matrix (ECM) pathways, resulting in increased ECM signaling and collagen deposition [49]. Furthermore, the study revealed significant differences in macrophage gene expression, suggesting that SARS-CoV-2 induces a more fibroproliferative response compared to the more exudative inflammatory response seen in H1N1 infection [49]. Wendisch et al. reported significant similarities between profibrotic monocyte-derived macrophage populations in COVID-19 and IPF [50]. Macrophages from patients with IPF and COVID-19 were both found to express fibrosis-associated genes including Secreted Phosphoprotein 1 (SPP1), transforming growth factor beta 1 (TGFB1), transforming growth factor beta-induced (TGFBI), legumain (LGMN), and C-C Motif Chemokine Ligand 18 (CCL18) [50]. TGF-B1 is an important regulator of interstitial fibrosis and BALf from patients with COVID-19 has been shown to be enriched with TGF-B1+ T-regulatory cells and CD14 cells [51]. Additionally, TGF-beta may orchestrate a sustained maladaptive immune reaction following COVID-19 infection with a resultant plasmablast shift to IgA2 expression [52]. CD163+ peripheral blood monocytes have been shown to be increased in patients with severe COVID-19. Interestingly, this subset of pro-fibrotic CD163+ macrophages has been shown to overlap with pro-fibrotic macrophages found in IPF [50]. Collectively, these findings suggest that COVID-19 infection may result in macrophage reprogramming towards a pro-fibrotic phenotype.

### 3.3. Telomere Shortening

There is an association between increasing age and higher COVID-19 mortality, which led to interest in the molecular pathways underlying aging that contribute to the severity of COVID-19. One area of interest is in the region of repetitive DNA sequences at the end of the chromosomes (telomeres) and telomere length (TL) [53]. A single Centre study in Madrid investigated the impact of telomere length (TL) on COVID-19 outcomes in 88 patients aged 29–85 years [53]. The study found a significant inverse correlation between TL and patient age, as well as a statistically significant inverse correlation between mean TL and COVID-19 severity as assessed by DNA-based techniques [53]. They also showed patients with either a lower mean or higher percentage of short telomeres had higher COVID-19 severity scores [53]. In another prospective cohort study of 77 patients, a linear association was found between the percent predicted telomere length and the predicted risk of fibrotic-like abnormalities in hospitalised COVID-19 survivors. Each 10% decrease in age-adjusted telomere length was associated with a 1.35 higher risk of fibrotic changes, fully adjusted for covariates [54].

## 4. Symptom Burden Post COVID 19

Both the post hospitalisation COVID-19 (PHOSP-COVID) study and the lung injury COVID-19 study found that a significant proportion of COVID-19 survivors experience persistent symptoms even months after hospital discharge [55,56]. The PHOSP-COVID study assessed 1077 patients across the United Kingdom (UK) who were discharged with a confirmed diagnosis of COVID-19 [55]. The study demonstrated that 92.8% of patients experienced at least one persistent symptom after a median follow-up of 5.9 months, with breathlessness and fatigue being among the most common symptoms [55]. The lung injury COVID-19 study which assessed patients 12 months after COVID-19 infection found that only one-third of patients with both moderate and severe COVID (as defined by level of respiratory support) reported resolution of symptoms [56]. The PHOSP-COVID study highlighted predictors of poor recovery such as female sex, having greater than two co-morbidities, and severe illness (defined as requiring mechanical ventilation or other organ support) during the acute phase [55]. Notably, symptomatic patients may not necessarily have PCILD, as deconditioning and pulmonary vascular disease can also contribute to respiratory symptoms. As such, objective evidence from CT scans and lung function tests must be correlated to accurately determine the cause of respiratory impairment.

## 5. Radiological and Pulmonary Function Findings Post COVID

With data from the initial stages of the pandemic demonstrating evidence of fibrotic changes seen as early as 2 months post COVID [24,57,58], there was a clear need for longer-term follow-up to determine the radiological and functional evolution of COVID-19 survivors. Most notably, what is the expected evolution of the organising pneumonia pattern seen in the acute phase of illness?

One of the largest meta-analyses assessing post-COVID-19 parenchymal and lung function abnormalities over time analyzed 46 studies and found persistent inflammatory changes in a number of patients [10]. At a median follow-up of three months, 50% of patients demonstrated inflammatory changes defined as GGO or consolidation on CT imaging [10]. In contrast, 29% had fibrotic changes defined as either reticulation, lung architectural distortion, interlobular septal thickening, traction bronchiectasis or honeycombing [10]. The meta-regression analysis showed that time was significantly associated with reduced radiological sequelae, particularly for inflammatory changes and more slowly for fibrotic changes [10]. Estimates of diffusing capacity for carbon monoxide (DLCO) were 38% across the 70 included studies, and the meta-regression analysis suggested that estimates of impaired DLCO reduced over time [10].

The UKILD post-COVID-19 study analyzed the percentage involvement of the lung and found that 79.4% of patients had residual lung abnormalities greater than 10% at a median of 113 days post discharge [59]. GGO was again the predominant abnormality, affecting a mean of 25.5% of the lung, as opposed to reticulation affecting only 15.1%. Thirty-three patients had repeat CT imaging after a minimum of 90 days, and interestingly the involvement of lung reticulations and GGO did not significantly change on repeated imaging [59]. There was a greater risk of residual lung abnormalities in males and those over 60 years of age, as well as those with severe acute illness, percent predicted diffusing capacity for carbon monoxide (ppDLCO) less than 80%, and abnormal chest radiological findings [59]. Despite a relatively short follow-up of 6 weeks, 1 study of 20 post-COVID-19 high-dependency unit (HDU) care patients found persistent fibrosis in the majority of the patients; however, despite persistent radiological abnormalities, mean FVC and DLCO improved over the study period [60]. Furthermore, this study found no association between DLCO and lung fibrosis [60].

Studies with a longer duration help to shed more light on the evolution of PCILD; one such prospective longitudinal study followed 83 COVID-19 survivors from the Wuhan area for up to 12 months [61]. Most patients had lung function improvement; however, one-third demonstrated ppDLCO less than 80% at 12 months and a small number of patients 11% had percent predicted forced vital capacity (ppFVC) less than 80% [61]. Residual CT changes were seen in 65% of patients at three months, with 78% showing GGO, 34% showing septal thickening, and 33% showing reticular opacity [61]. At nine months, 20% of patients still had abnormal CT scans, but none showed definitive or established fibrosis, and none showed progressive changes. The predominant inflammatory pattern remained GGO, and there was no further improvement between nine and twelve months [61]. This study excluded patients who required mechanical ventilation or had a history of hypertension, diabetes, or obstructive lung disease, which may have resulted in selection bias, as these patients may be more likely to experience severe acute illness and have a different recovery trajectory. Other longer-duration studies conducted in both China and Europe have revealed persistent radiological abnormalities on CT imaging during the 12-month follow-up. Once again, GGO emerged as the prevailing observation in these studies [62,63,64]. While the European studies indicated a gradual improvement in DLCO over time, the Chinese cohort exhibited no such improvement. One study involving two experienced radiologists, visually analyzed and compared CT scans at six and twelve months classifying them as residual non-fibrotic and residual fibrotic using standard definitions and international guidelines [65]. It reported that for 46 patients with non-fibrotic residual abnormalities, these completely resolved in 26 cases and improved in the other 20; however, those with fibrotic changes remained unchanged at 12 months [65]. A recent scientific letter presented data from a cohort of 209 intensive care unit (ICU) COVID-19 patients from a single Centre in Spain with a 2-year follow-up [66]. They found recovery in lung function and exercise capacity over time but a persistent impairment in DLCO in 45.7%, with 18.7% classified as a moderate–severe impairment. More than half of follow-up CT scans demonstrated some persistent abnormalities (39.2% reticular lesions and 12.7% classified as fibrotic) [66]. This fibrotic pattern was more frequent in intubated patients [66].

Drawing from the established associations between acute disease severity, radiographic abnormalities, and the findings from autopsies of mechanically ventilated COVID-19 patients revealing heightened pneumocyte hyperplastic and metaplastic alterations [67], several studies have investigated the effect of disease severity on post-COVID radiological abnormalities [56,68]. The lung injury COVID-19 study included a subset of 79 patients who underwent follow-up CT scans 12 months post-discharge stratified by level of respiratory support [56]. They found that a higher proportion of patients in the severe cohort (those requiring non-invasive ventilation, high-flow nasal cannula, or invasive mechanical ventilation) demonstrated persistent radiological abnormalities compared to the moderate group (those requiring supplemental oxygen by mask or nasal prongs) [56]. Specifically, 87% of the severe group demonstrated traction bronchiectasis, 69% demonstrated coarse reticulation, and 71% demonstrated GGO, compared to 37%, 32%, and 20%, respectively, in the moderate group [56]. This study also looked at lung function between the severity groups at ≥10-month follow-up, with functional normalization more common in the moderate group than in the severe group (64.1% vs. 50.6%, *p* < 0.001) [56]. However, 42.4% of these 153 patients demonstrated persistent impaired DLCO at 12 months [56]. Multivariate analysis revealed that age, percent predicted forced expiratory volume in one second (FEV1), and dyspnea score during follow-up were independent risk factors associated with impaired ppDLCO [56]. A small study of both ICU and ward-based COVID-19 survivors assessed a subset of 75 patients with lung function and CT imaging at four months [68]. In this subset, 21% had evidence of fibrosis (traction bronchiectasis, architectural distortion, or honeycombing), and 44% had GGO [68]. All patients who had acute imaging from admission showed temporal improvement on follow-up [68]. Out of the total cohort, only 7% demonstrated persistent fibrosis at the 4-month follow-up [68]. The ICU cohort had a statistically significant increase in radiological changes compared to the ward-based cohort [68]. Furthermore, in the ICU cohort, ppDLCO negatively correlated with increased length of stay and higher maximum inspired oxygen Fi02. The lowest ppDLCO was observed in patients with fibrotic changes [68]. The PHOSP-COVID study also found correlations between acute illness severity and lung function impairment, of the total cohort 33.3% of patients had ppFVC less than 80% and 34.3% of patients had ppDLCO less than 80% [55]. Impairments were more prevalent in the severe cohort of patients for both parameters. Additionally, the study found that DLCO appeared to be worse in patients who required mechanical ventilation during their acute illness [55].

With real-world studies being conducted during varying pandemic waves, with diverse management strategies and wide-ranging follow-up periods drawing generalizable conclusions about the novel entity that is PCILD is difficult. Compounding this challenge is the absence of a standardized definition of PCILD. This is reflected in several meta-analyses on post-COVID-19 ILD, demonstrating considerable heterogeneity [69,70]. One such meta-analysis categorized CT findings into fibrotic and non-fibrotic appearances using the Fleischner Society glossary [69]. This analysis found organising pneumonia or non-specific interstitial pneumonia (NSIP) patterns were most commonly reported [69]. Interestingly, only a handful of studies reported fibrotic indicators like traction bronchiectasis or bronchiectasis; notably, the presence of honeycombing was a rare occurrence, with a pooled prevalence as low as 0.2% [69]. Another meta-analysis that assessed lung function and chest CT appearance six to twelve months post COVID found impaired DLCO as the most common abnormality on follow-up PFTs. The pooled prevalence of pulmonary fibrosis in this meta-analysis was 32%, the follow-up duration did not have an impact on the prevalence of pulmonary fibrosis [70].

It is apparent that while COVID-19 survivors may experience persistent radiological abnormalities, impaired lung function, and symptom burden even after recovery from the acute phase of the illness, a proportion of patients demonstrate at least some improvement in DLCO over time (See Table 1).

Emerging risk factors for post-COVID-19 ILD include:

- Severity of illness: those who required non-invasive or mechanical ventilation during their acute illness were more likely to develop residual lung abnormalities, including ground-glass opacities, reticulation, and fibrosis, which can persist for several months after discharge;
- Age: older patients, particularly those over 60 years of age, were more likely to experience persistent lung abnormalities and impaired lung function;
- Male sex: male patients were at higher risk of residual lung abnormalities after COVID-19 hospitalisation.

## 6. Follow Up

**Figure 1:**
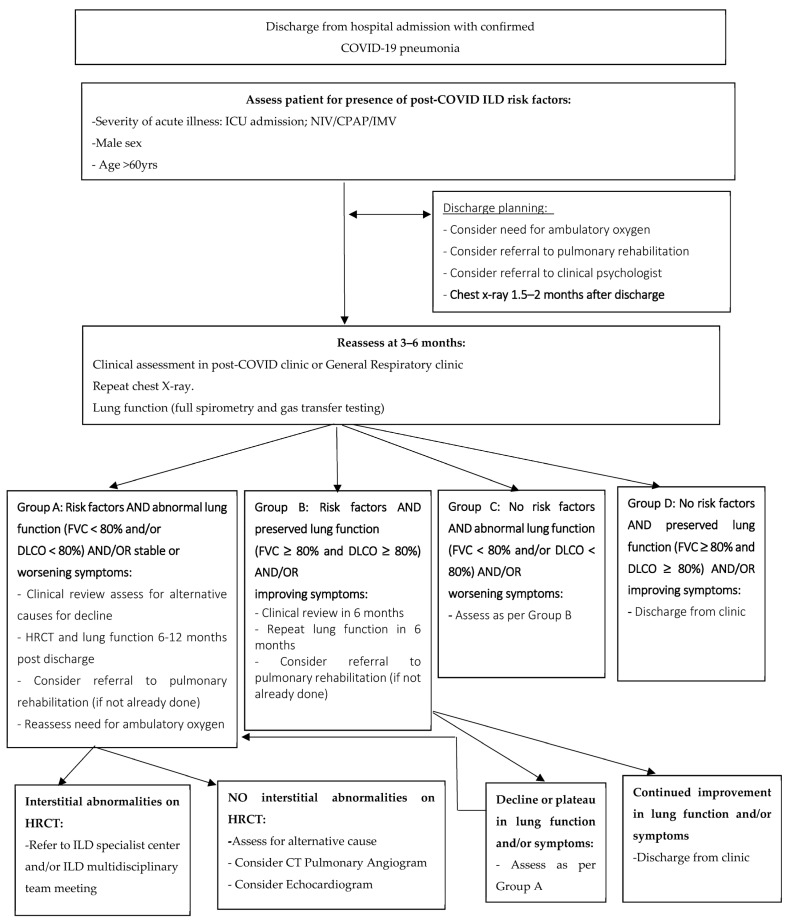
Proposed flowchart for post-discharge follow-up of patients hospitalized due to COVID-19 pneumonia.

The level of uncertainty surrounding PCILD can be difficult to manage for both clinicians and patients; however, risk assessments may provide a way to customize follow-up and referral to specialist ILD centers for high-risk cases. We have created an algorithm (Figure 1) to help clinicians identify and triage patients with possible PCILD, providing a practical tool for managing this emerging condition. Symptom management and quality of life should play a central role in any management strategy for cases where post-COVID-19 pulmonary sequelae are present. This holds significant relevance, given that COVID-19 pneumonia frequently leads to extended hospitalisations and ICU admission [19]. The inflammatory and hypoxic conditions inherent in acute COVID-19 ultimately contributed to diminished exercise capacity, deconditioning, increased frailty, and altered breathing patterns which can be seen as part of the post-COVID-19 sequelae [19]. Present approaches to address post-COVID-19 breathlessness have been adapted from practices for post-ICU care and chronic respiratory conditions; however, there remains a need for tailored rehabilitation strategies specific to the post-COVID-19 context [19].

## 7. Management Strategies for Post-COVID ILD

No current standard of care treatment for PCILD exists; however, numerous randomized clinical trials (RCT) are ongoing to address this paucity of evidence. Given that organising pneumonia (which is known to be steroid responsive) is the most common radiological pattern during acute and follow-up scans, much interest has been invested in corticosteroid treatment [18,71]. Notably, the efficacy of dexamethasone in lowering mortality rates among severe COVID-19 cases has underscored the significance of such treatment avenues. In a study by Myall et al. involving 30 patients diagnosed with PCILD through a multidisciplinary team approach and displaying an organising pneumonia pattern, the administration of prednisolone (at a dosage of 0.5 mg/kg) resulted in improvements in lung function, symptoms, and radiological appearances [18]. The STERCOV-ILD (NCT04988282) phase 4 open-labelled RCT recently reported good clinical and functional responses to methylprednisolone 0.5 mg/kg/day for 4 weeks; however, there were no significant differences when considering a greater than 90% resolution of lesions on CT [72]. In addition, the COLDSTER trial reported that high-dose prednisolone was not associated with any improvement in clinical, radiological, or quality-of-life outcomes when compared to lower-dose prednisolone; however, this trial lacked a placebo arm [73]. Consequently, the role of corticosteroid therapy in PCILD remains to be elucidated.

Tocilizumab [6] and Baricitinib [7] immunomodulatory agents used in acute COVID-19 are being considered for post-COVID ILD due to their efficacy in other inflammatory conditions [74,75]. However, the RECOVERY arms for both medications [6,7] did not assess radiographic or functional outcomes for either drug. The role of immunosuppressive therapies in PCILD is also not clear considering that the PANTHER-IPF study found an increased risk of death and hospitalisation in IPF patients treated with immunosuppression (as a combination of azathioprine, prednisone, and N-acetylcysteine) [76]. Nevertheless, early in vivo studies have suggested that inter-leukin-6 and Janus kinase (JAK) inhibition may be promising areas of interest in IPF [77,78] and both Tocilizumab and Baricitinib are drugs that target these pathways.

There is understandably much interest in the role of antifibrotic therapy in COVID-19 and there are already several studies underway investigating the role of both Pirfenidone (FIBRO-COVID, NCT04607928) and Nintedanib (NINTECOR, NCT04541680) for PCILD. Notably, the PINCER trial (NCT04856111) will compare these established antifibrotic treatments in a phase 4 head-to-head study evaluating the safety and efficacy of Pirfenidone versus Nintedanib. Several case reports have looked to assess the role of antifibrotics in acute COVID-19 illness with variable results [79]. A recently published study randomized symptomatic post-COVID-19 patients (at 12 weeks post discharge) to either Nintedanib or Pirfenidone. They reported improvements in lung function, exercise tolerance, oxygen saturation, and radiological scoring [80]. This study lacked a control arm and reported CT scores as opposed to distinct patterns or fibrotic/non-fibrotic features. There is still a considerable amount to uncover regarding the role of antifibrotics in PCILD. Additionally, the anticipated trajectory of this condition remains an area of exploration, especially considering that PCILD has not demonstrated a progressive nature up to this point.

## 8. Conclusions

Emerging data from post-COVID-19 follow-up studies suggest that interstitial lung disease (ILD) is a relatively common consequence of SARS-CoV-2 infection with organising pneumonia as the predominant radiological pattern. It appears that GGO appearances and DLCO impairment demonstrate some improvement over time, while current evidence suggests a more static picture in a smaller minority with fibrotic changes. [10,55,56,59,60,61,68]. Time will only tell the true behavior of this new disease entity with significant genetic and pathophysiological overlap with IPF. Indeed, whether these appearances should be dichotomized into fibrotic versus inflammatory remains an area of debate [38]. Several similar review articles have also attempted to summarize the ever-growing literature discussing PCILD and drawn similar conclusions to those presented here [81,82,83]. To our knowledge, this is the first review suggesting a follow-up algorithm to identify and stratify patients with possible PCILD. This approach builds upon the groundwork laid by George et al., who previously outlined a framework for the respiratory follow-up of post-COVID-19 patients [84]. Their framework advocated for the referral of patients displaying any evidence of ILD to specialized ILD services. In anticipation of a considerable proportion of patients manifesting residual lung abnormalities following COVID-19 but with some functional and/or clinical improvement, our suggested pragmatic algorithm aims to not only direct the most critical cases toward specialized care but also to assist non-ILD clinicians and health professionals in managing patients with post-COVID pulmonary sequelae. Identifying a consensus diagnosis for PCILD can only help to further our understanding of this disease. Evidence is also likely to evolve as more studies encompassing or comparing patients from later stages of the pandemic (with different variants and acute illness management) are assessed. Further research is needed to understand the natural history of COVID-19-related lung disease, identify factors that may predict long-term respiratory outcomes, and develop appropriate monitoring and care strategies for COVID-19 survivors. As clinicians await more data and clarity, it is evident that patients with PCILD will undoubtedly benefit from MDT discussion, symptom management, pulmonary rehabilitation, and phenotyping according to treatable traits.

## Figures and Tables

**Table 1:** Summary of published studies on radiological and lung function sequelae following acute COVID-19 illness.

**Study**	**Type of Study**	Dataset	Duration of Follow-Up	Radiological Findings	Lung Function	Radiological and/or Functional Improvement	Comments
Fabbri et al. [10]	Systematic review and meta-analysis	70 studies for quantitative synthesis. 46 studies included in meta-analysis of radiological sequelae	3 months (median)	Inflammatory sequelae estimated in 50%, fibrotic sequelae estimated in 29%.	Impaired DLCO estimated at 38% of lung function tests	Yes—meta-regression analysis showed time was significantly associated with reduced radiological inflammatory changes; however, more slowly for fibrotic changes.	High heterogeneity, differences in study cases.
Yang et al. [24]	Retrospective	China (*n* = 166)	56 days median (IQR 51–63)	46% cohort CT evidence of fibrotic changes (parenchymal bands 76%, traction bronchiectasis 38%, lung distortion 25%, and honeycombing 9%).	N/A	Yes—of 76 patients with evidence of fibrosis on peak CT 37% classified as severe as opposed to follow-up scan where 59% classified as minimal.	77% of cases classified as severe or critical acute cases.
Vargas et al. [56]	Prospective clinical cohort	Spain (*n* = 305): moderate, 162; severe, 143	12 months	17.8% CXR abnormalities at 12-month follow-up; 79 of cohort completed CT at 12 months CT scans showed evidence of fibrosis including parenchymal bands, coarse reticular patterns, irregular interfaces, and traction bronchiectasis in ~50% of severe cohort and ~25% of moderate cohort.	42.4% of patients with decreased pulmonary diffusion 10 months after infection onset	No—higher rate of CXR abnormalities at 5 month follow-up.	ppDLCO significantly lower in patients with abnormalities on radiology imaging.
Marvisi et al. [57]	Retrospective	Italy (*n* = 90)	8 week follow up; CT at 60 days post admission	54.4% of patients developed diffuse GGO; 46.6% developed both GGO and consolidations; fibrotic appearances in 25.5% typically NSIP pattern.	DLCO noted to be lower in those classified with fibrotic CT appearances. (DLCO/VA 58% ± 13)	Yes—area of GGO/consolidation decreased/disappeared in 50%.	Risk factors for fibrosis: male sex, smoking habit, and comorbidities.
Zhou et al. [58]	Prospective cohort study	China (*n* = 216) Stratified patients by acute illness severity	~4 months	85.1% of severe cohort and 68.0% of mild/moderate cohort had residual CT abnormalities at follow-up; most commonly GGO followed by strip-like fibrosis.	Significantly reduced TLC%, RV%, and DLCO% values in severe and mild/moderate groups	Yes—CT improvement at follow-up noted in both groups but was higher in the mild/moderate group.	Significant negative correlation between the extent of CT abnormalities and TLC% and RV% at the 3-month follow-up, but a weaker correlation with DLCO% (all *p* < 0.05).
Stewart et al. [59]	Interim analysis of a prospective longitudinal cohort study	UK (*n* = 3700 255 participants with linked CT scans)	Follow-up CT at 113 days median (IQR 69–166)	Residual lung abnormalities were estimated in up to 11% of people discharged after COVID-19; 79.4% had more than 10% involvement of residual lung abnormalities; GGO affected a mean of 25.5 % of the lung, reticulation a mean of 15.1 %, with residual abnormalities involved a mean of 40.6% of the lung.	8% of total cohort demonstrated ppFVC < 80% and 4.8% of total cohort demonstrated ppDLCO < 80% (N.B. data missing for 71% and 86%, respectively)	Minimal—33 people with repeat CT at 90 days in paired analysis, the overall change in residual lung abnormalities was −3.62%.	Risk factors: ppDLCO less than 80%, abnormal CXR, and severe illness on admission.
Santus et al. [60]	Prospective cohort study	Italy (*n* = 20)	6 weeks post discharge	Proportion of CT scans showing multifocal GGO increased from 30% during admission to 80% at 6-week follow-up (*p* = 0.002).	Mean FVC % predicted: 87.4% Mean DLCO %predicted: 67.2%	Yes—statistically significant improvement in FVC and DLCO improved from hospitalisation	DLCO impairment correlated with disease severity and was associated with abnormal CT both during acute phase and at 6-week follow-up.
Wu et al. [61]	Prospective, longitudinal, cohort study	China (*n* = 83)	3, 6, and 12months	Radiological changes persisted in 20 (24%) patients at 12-month follow-up; with predominant GGO. At 12 months, none of the HRCT scans showed evidence of established fibrosis and none showed evidence of progressive interstitial changes.	27 patients had impaired DLCO (ppDLCO < 80%) at 12-month follow-up. Median ppDLCO: 88% at 12-month follow-up. Median FVC 92% predicted at 12-month follow-up	Yes—most patients showed an improvement in their pulmonary function at each timepoint of 3 months, 6 months, and 12 months.	On univariate analysis, risk factors associated with abnormal HRCT at 12 months included length of hospital stay, peak HRCT scores during hospitalisation, and receiving HFNC or NIV.
Faverio et al. [62]	Prospective, observational study	Italy (*n* = 287)	12 months	Mild non-fibrotic radiological abnormalities in 66% of cohort. Majority demonstrated GGO 51%), only 1% demonstrated honeycombing.	Almost 40% of patients showed DLCO impairment (classified as mild)	Yes—DLCO showed a trend of improvement between 6- and 12-month follow-up regardless of level of respiratory support during acute illness.	IMV identified as risk factors for pathological HRCT.
Tarraso et al. [63]	Prospective, observational cohort study	Spain (*n* = 284 completed evaluation)	12 months	27.4% of total cohort had an abnormal CT at 12 months; GGO found in 45.5%, reticular pattern in 34%, traction bronchiectasis in 30.8%, and parenchymal bands in 33.4%. In total, fibrotic-like sequelae were in 22.7% of total cohort at 12 months.	At 12-month follow-up, however, impaired DLCO in 39.8% unrelated to severity. ppFVC < 80% was 6.7% at 12-month follow up	Yes—improving trend in DLCO over follow-up. Percentage of GGO on CT decreased over time. (Comparing 2 month to 12 month CT).	Only risk factor identified for fibrosis was appearance of admission CT.
Huang et al. [64]	Ambi-directional cohort study	China (*n*= 1276); patients were categorized by acute illness severity based on level of respiratory support	6 and 12 months; 128 patients completed CT at 12 months	At 12 months, 39%, 40%, and 87% of the mild, moderate, and severe groups, respectively, demonstrated at least one abnormal CT pattern, with GGO the most common finding in all cohorts; 12 months. The proportion of patients with abnormal CT decreased significantly from 6 months to 12 months in all severity groups.	ppDLCO < 80% at 12 months was found in 23%, 31%, and 54% of the mild, moderate, and severe groups respectively.	Yes—the proportion of patients with abnormal CT decreased significantly from 6 months to 12 months in all severity groups with GGO decreasing over time; however, the proportion of patients in the several groups with interlobular septal thickening significantly increased over time.	Most patients demonstrated good physical and functional recovery. However, lung diffusion impairment and radiographic abnormalities persisted in some especially those who were critically ill during acute illness.
Besutti et al. [65]	Retrospective cohort study	Italy (*n* = 405)	5–7 months	Residual non-fibrotic abnormalities found in 37.5%—most frequently assigned radiological pattern as non-fibrotic NSIP (103/152). Residual fibrotic abnormalities reported in 6.9%.	N/A	Yes—resolution in 55.6% when assessed by 2 radiologists (cumulative experience 25 years).	
González et al. [66]	Prospective cohort study of ICU patients	Spain (*n* = 180)	6, 12 and 24 months	At 24 months, 53.9% of patients with residual abnormalities classified as 39.2% reticular lesions and 12.7% fibrotic involvement.	At 24 months 45.7% with DLCO impairment in DLCO (18.7% of them classified as moderate to severe	Yes—over 2 years, patients showed a progressive recovery of lung function and exercise capacity. There was also evidence of radiological improvement evidenced by reduction in severity score and number of lobes affected.	Patients who required IMV showed worse DLCO values compared to non-intubated patients.
Robey et al. [68]	Retrospective service evaluation	UK (*n* = 221)	4 months	Completed CT scans on 72 patients at an average of 18 weeks post discharge. Demonstrating evidence of persistent ground glass opacities in 44% and fibrosis in 21% (equating to 7% of the entire cohort).	Most common lung function abnormality: decreased DLCO	N/A	DLCO correlated negatively with length of stay and with maximum inspired FiO_2_.
Bocchino et al. [69]	Systematic review and meta-analysis	14 Studies	12 months	The pooled estimate of 1-year chest CT lung sequelae is 43.5%; GGO most reported nonfibrotic change (range 2.4–67.7%). The estimated range of fibrotic traction bronchiectasis/bronchiolectasis was 1.6–25.7%. Honeycombing was unrepresented (range, 0–1.1%; three studies)	N/A	N/A	High heterogeneity demonstrated. No study characteristics emerged to determine heterogeneity, with root causes still unknown.
Lee et al. [70]	Systematic review and meta-analysis	30 studies	Between 6 and 12 months	On follow-up chest CT, pooled prevalence of GGO was 34%, pooled prevalence of pulmonary fibrosis was 32%, and the prevalence did not decrease over time.	Impaired DLCO most common abnormality on PFT (pooled prevalence 35%); reduced FVC forced vital capacity was less frequent (pooled prevalence 8%)	Yes—FVC impairment less prevalent at 12 months than at 6 months; however, follow-up duration did not have an impact on the prevalence of pulmonary fibrosis.	Significant between-study heterogeneity. Severity of index infection was associated with the prevalence of impaired DLCO and pulmonary fibrosis.

Abbreviations; DLCO: diffusion of the lung for carbon monoxide, IQR: interquartile range, CXR: chest X-ray, ppDLCO: percent predicted diffusing capacity for carbon monoxide, GGO: ground-glass opacity, NSIP: non-specific interstitial pneumonia, DLCO/VA: diffusion of the lung for carbon monoxide/alveolar volume, TLC: total lung capacity, RV: residual volume, ppFVC: percent predicted forced vital capacity, HFNC: high flow nasal cannula, NIV: non-invasive ventilation, IMV: invasive mechanical ventilation, FiO2: fraction of inspired oxygen.

## Data Availability

This is a review article and, therefore, no new data were created.
